# Development of the H3N2 influenza microneedle vaccine for cross-protection against antigenic variants

**DOI:** 10.1038/s41598-022-16365-2

**Published:** 2022-07-16

**Authors:** Yura Shin, Jeonghun Kim, Jong Hyeon Seok, Heedo Park, Hye-Ran Cha, Si Hwan Ko, Jae Myun Lee, Man-Seong Park, Jung-Hwan Park

**Affiliations:** 1 Department of BioNano Technology, Gachon University, Seongnam, Republic of Korea; 2 Department of Microbiology, Institute for Viral Diseases, Chung Mong-Koo Vaccine Innovation Center, College of Medicine, Korea University, 73 Goryeodae-ro, Seongbuk-gu, Seoul, 02841 Republic of Korea; 3 Department of Microbiology and Immunology, Institute for Immunology and Immunological Diseases, Yonsei University College of Medicine, Seoul, 03722 Korea

**Keywords:** Vaccines, Biomedical engineering

## Abstract

Due to the continuously mutating nature of the H3N2 virus, two aspects were considered when preparing the H3N2 microneedle vaccines: (1) rapid preparation and (2) cross-protection against multiple antigenic variants. Previous methods of measuring hemagglutinin (HA) content required the standard antibody, thus rapid preparation of H3N2 microneedle vaccines targeting the mutant H3N2 was delayed as a result of lacking a standard antibody. In this study, H3N2 microneedle vaccines were prepared by high performance liquid chromatography (HPLC) without the use of an antibody, and the cross-protection of the vaccines against several antigenic variants was observed. The HA content measured by HPLC was compared with that measured by ELISA to observe the accuracy of the HPLC analysis of HA content. The cross-protection afforded by the H3N2 microneedle vaccines was evaluated against several antigenic variants in mice. Microneedle vaccines for the 2019–20 seasonal H3N2 influenza virus (19–20 A/KS/17) were prepared using a dip-coating process. The cross-protection of 19–20 A/KS/17 H3N2 microneedle vaccines against the 2015–16 seasonal H3N2 influenza virus in mice was investigated by monitoring body weight changes and survival rate. The neutralizing antibody against several H3N2 antigenic variants was evaluated using the plaque reduction neutralization test (PRNT). HA content in the solid microneedle vaccine formulation with trehalose post-exposure at 40℃ for 24 h was 48% and 43% from the initial HA content by HPLC and ELISA, respectively. The vaccine was administered to two groups of mice, one by microneedles and the other by intramuscular injection (IM). In vivo efficacies in the two groups were found to be similar, and cross-protection efficacy was also similar in both groups. HPLC exhibited good diagnostic performance with H3N2 microneedle vaccines and good agreement with ELISA. The H3N2 microneedle vaccines elicited a cross-protective immune response against the H3N2 antigenic variants. Here, we propose the use of HPLC for a more rapid approach in preparing H3N2 microneedle vaccines targeting H3N2 virus variants.

## Introduction

Active ingredients with a large molecular weight, such as a vaccine, can pass through the stratum corneum without pain and can be delivered into the dermal layer by using microneedles with a length of 100–1000 μm^1,2^. Due to the presence of immune cells within the dermal layer, vaccine delivery using microneedles has been actively studied^3–8^. In particular, influenza microneedle (MN) vaccines are being preclinically and clinically studied and have been found to produce immunological efficacy similar to that provided by intramuscular (IM) administration^9–13^.

Seasonal influenza viruses cause an epidemic in local regions which then spreads globally and therefore recognition of seasonal viruses at an early stage is important in determining the virus that will be prevalent in a year^14^. However, the efficacy of a seasonal influenza vaccine is greatly reduced due to antigenic variation as well as when vaccine strain does not match with circulating strain^15–17^. In particular, seasonal H3N2 influenza vaccines show a low level of effectiveness due to antigenic variation^18^. Thus, cross-protection provided by the H3N2 vaccine is essential due to the mutating frequency of the H3N2 virus^19–26^.

Two proteins; hemagglutinin and neuraminidase are present on the surface of influenza A virus (IAV) and the quantification of the influenza vaccine antigen is determined by measuring the concentration of the HA protein. Currently, single radial immunodiffusion (SRID) assay or enzyme-linked immunosorbent assay (ELISA) are commonly used to measure the concentration of the influenza vaccine antigen. However, these methods require standard antigens and antibodies provided by the National Institute for Biological Standards and Control (NIBSC) along with a 2–3 month preparation period, which may be a limiting factor in the manufacturing of vaccines. It was reported that the HA content measured by high performance liquid chromatography (HPLC) for H1N1- and H3N2-subtype viruses was comparable to that of SRID and ELISA assays^27–29^.

**Figure 1:**
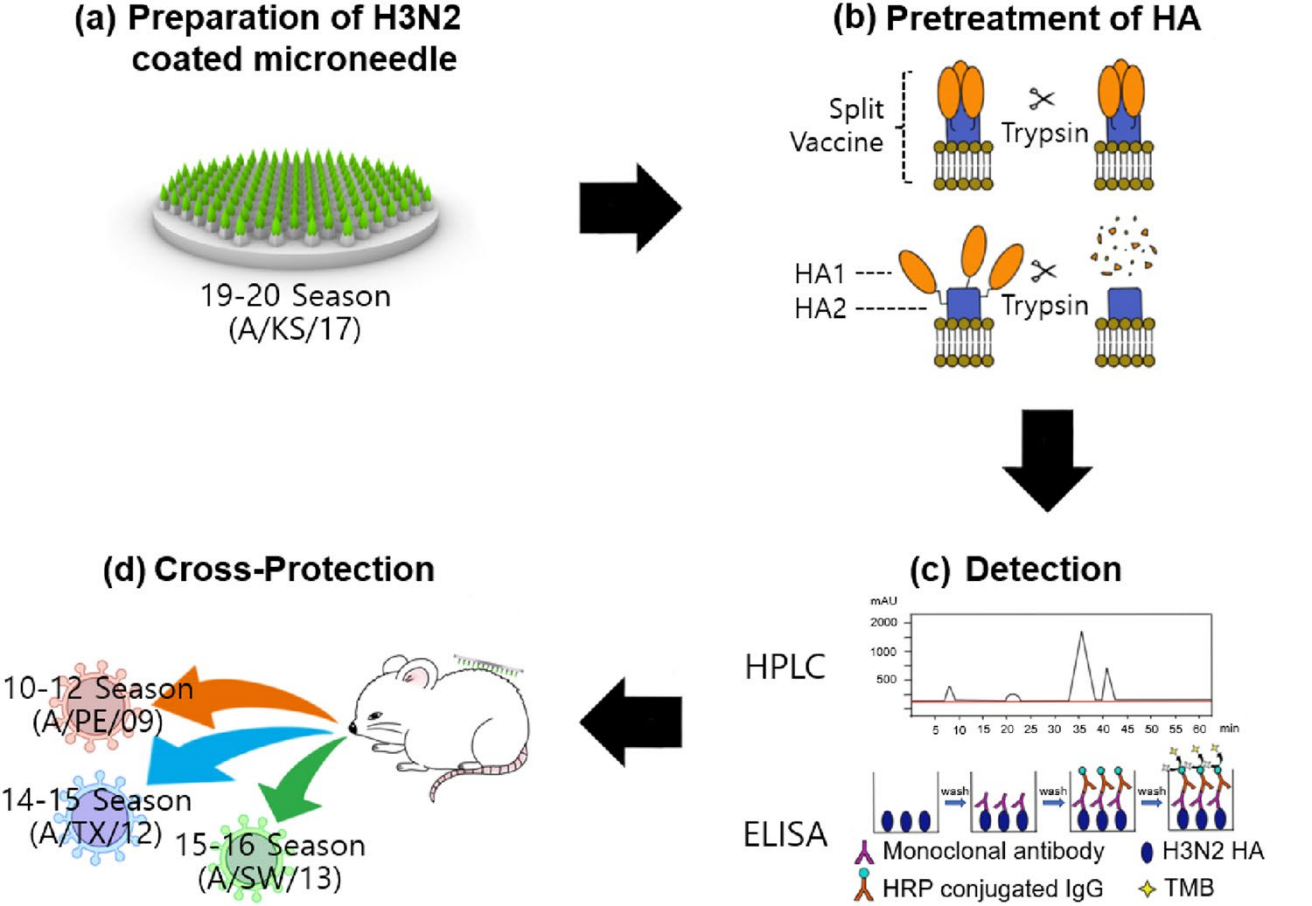
Schematic diagram of H3N2 microneedle vaccine development based on HPLC analysis of HA for cross-protection of influenza H3N2 virus. (**a**) Preparation of 19–20 A/KS/17 H3N2 microneedle vaccine, (**b**) pretreatment of HA, (**c**) establishment of HPLC-based analysis of HA content of H3N2 vaccine stability and comparison with ELISA, and (**d**) observation of cross-reactivity immune response against 10–12 A/PE/09, 14–15 A/TX/12, and 15–16 A/SW/13 H3N2 viruses in animal experiments.

In order to prepare a microneedle vaccine to target a virus that mutates frequently like H3N2, a measurement for the amount of active antigen is required in order to load an accurate predetermined amount of vaccine into the microneedle. However, if several months are needed for antibody preparation, the preparation of microneedle vaccines as well as related stability studies become increasingly difficult. Therefore, measurement of antigen activity without the standard antibody facilitates the preparation process of microneedle vaccines. In this study, as shown in Fig. 1a, H3N2 microneedle vaccines were prepared using HPLC measurement of HA content, and because the H3N2 virus is highly mutable, cross-protection of H3N2 microneedle vaccines was observed. Measurement of HA content is important for the preparation of H3N2 microneedle vaccines as it can be used to detect a possible denaturation of the H3N2 vaccine. The HA content of H3N2 and the stability of the formulation were measured using HPLC because HPLC analysis does not require the H3N2 antibody (Fig. 1b). H3N2 microneedle vaccines were prepared with the 2019–20 seasonal A/Kansas/14/2017 influenza H3N2 vaccine (19–20 A/KS/17). Cross-protection against 2010–12 seasonal A/Perth/16/2009 influenza H3N2 virus (10–12 A/PE/09), 2014–15 seasonal A/Texas/30/2012 influenza H3N2 virus (14–15 A/TX/12), and 2015–16 seasonal A/Switzerland/9715293/2013 influenza H3N2 virus (15–16 A/SW/13) was also evaluated (Fig. 1c). The cross-protective response of the microneedle vaccination was compared with that of conventional IM vaccination.

## Results and discussion

### Geometries of H3N2 microneedle vaccine

**Figure 2:**
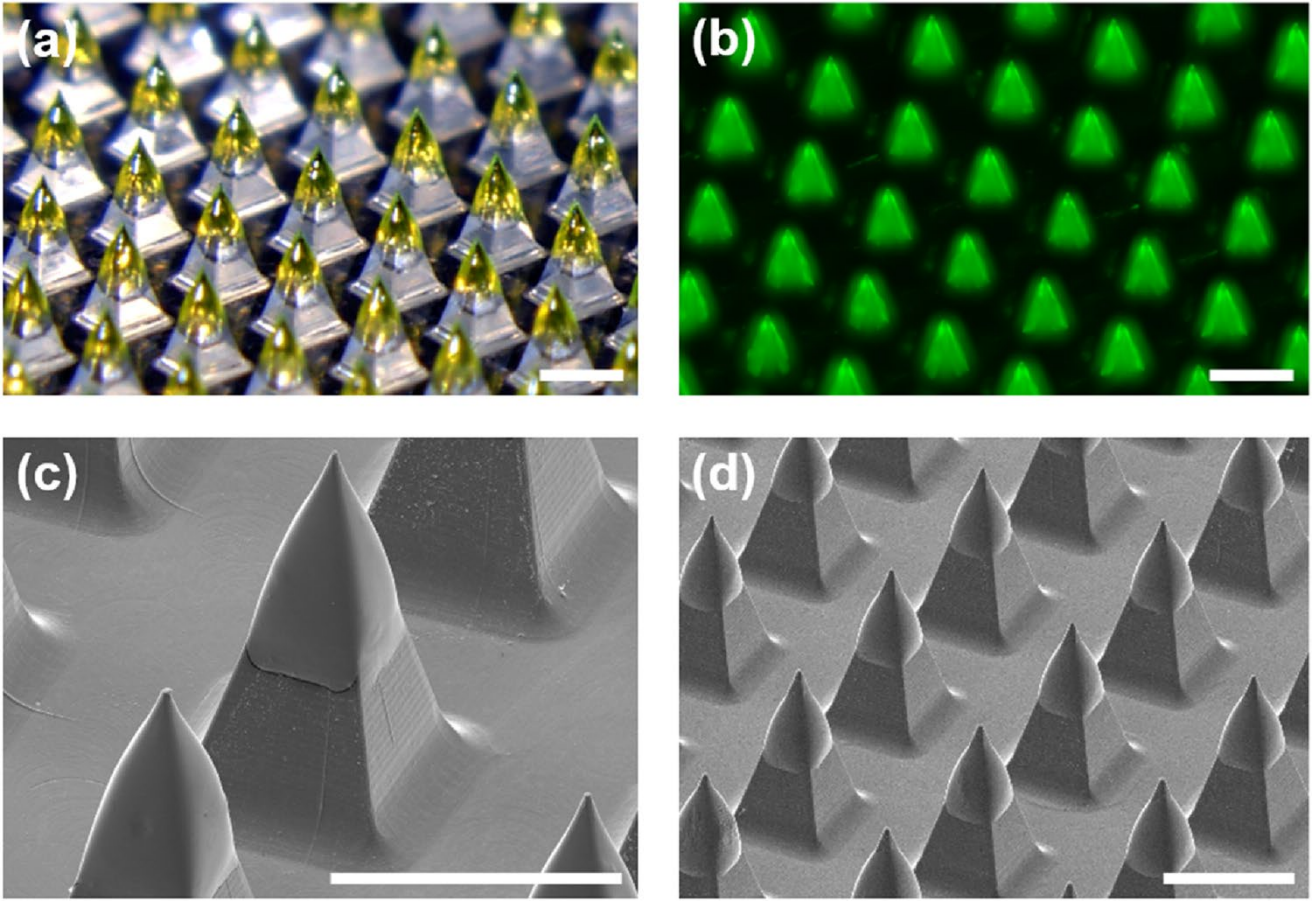
Images of coated microneedles. (**a**) Optical microscopic image (scale bar = 500 μm) and (**b**) fluorescence image of FITC-dextran coating layer on microneedles (scale bar = 500 μm). (**c**) Enlarged SEM image of H3N2 microneedle vaccine. (**d**) SEM image of H3N2 microneedle vaccine array (scale bar = 500 μm).

The uncoated H3N2 microneedle vaccines were made of PLA, an FDA-approved polymer. When the antigen content of the MN-Tre20 and MN-Tre100 groups was analyzed by Bradford protein assay, the loading amounts of HBsAg were 3.1 ± 0.3 and 3.1 ± 0.2 μg/array, respectively. The manufacturing process of H3N2 influenza microneedles requires repeated measurements of many samples during the optimization and manufacturing processes. For the large number of samples, protein concentration measurement provided fast quantitative analysis. The solid H3N2 vaccine formulation contains stabilizers and excipients in addition to the vaccine. Most of the solid formulation consists of these additives, and the vaccine is uniformly distributed within the formulation. In this study, the protein content of the solid formulation was 1.4% and 0.66% for Tre-20 and Tre-100, respectively. Therefore, if the amount of coated formulation is uniform, the vaccine content is uniform. In this study, the standard deviation in vaccine amount between microneedles was 10% or less. Also, as shown in the optical image in Fig. 2a and the fluorescence microscope image in Fig. 2b of microneedles coated with the FITC-dextran formulation, there was a uniform coating layer over all tips of the microneedles in the array. SEM images in Figs. 2c,d, show that the coating layer covered approximately one third of the microneedles from the tip, to allow for delivery into the skin even if the microneedles were only partially inserted. The distribution of the coating layer near the end of the tips is helpful for proper delivery of the vaccine.

### Puncture performance of coated microneedles

**Figure 3:**
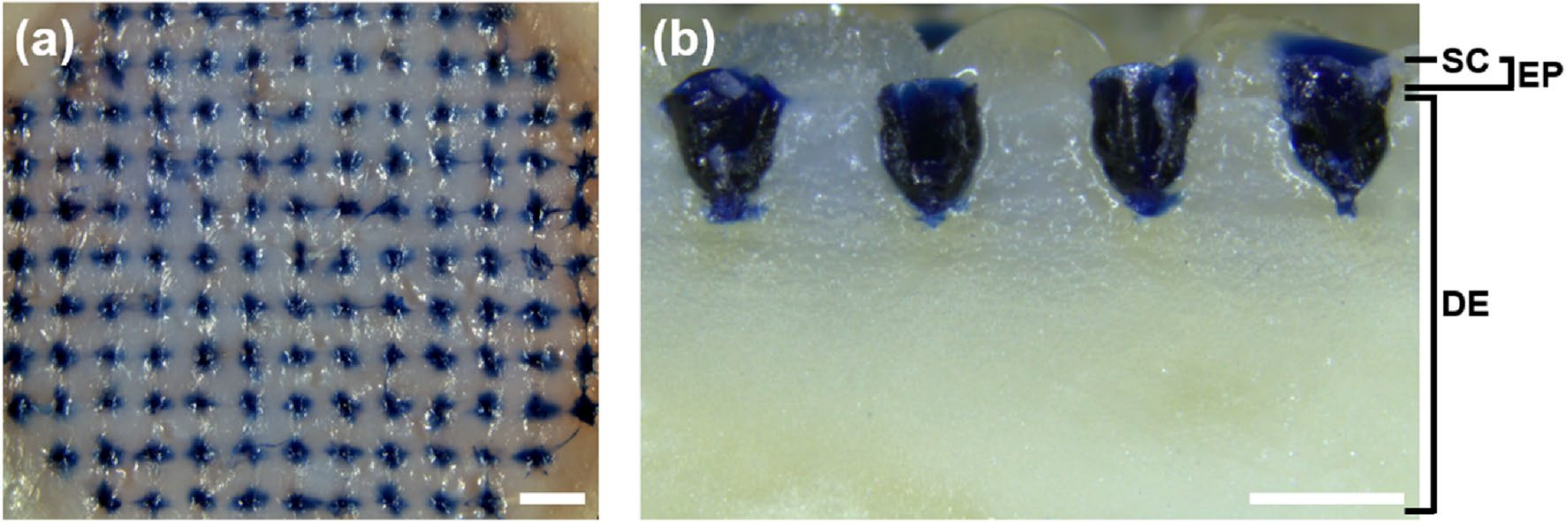
(**a**) Optical image of the porcine skin surface after administration and removal of trypan blue microneedles (Scale bar = 1 mm). (**b**) Optical image of a cross-section of the microneedles passing through the blue dots (Scale bar = 500 μm). ‘SC’, ‘EP’, and ‘DE’ refer to stratum corneum, epidermis, and dermis, which are components of the skin layer, respectively. Trypan blue containing the vaccine was transferred to the epidermis and dermis of the skin.

All microneedles were successful in penetrating the skin, and the coating layer was delivered into the skin (Fig. 3a). Observing the cross-section of the trypan blue dots showed that the insertion depth of the coating layer into the skin was 600 μm and the trypan blue layer was distributed through the epidermis and dermis, as shown in Fig. 3b. Thus, PLA microneedles had sufficient mechanical strength to penetrate the stratum corneum and successfully delivered the coating layer into the skin. The microneedle system used in this study involves coated microneedles. Compared to other types of microneedles, our microneedle type is suitable for vaccine delivery because the manufacturing temperature can be controlled^11^ and the mechanical strength for successful insertion is provided by the microneedle shafts of coated microneedles^30^. Thus, mechanical strength should not be considered for the design of the formulation and various kinds of materials can be used as additives for improving the stability of the vaccine and increasing immune efficacy.

### Ex vivo delivery efficiency of coating layer

The amount of residual FITC-dextran on the surface of the microneedles after administration to porcine skin for 30 min and removal was 18% of the total amount applied to the microneedles. The amount of FITC-dextran remaining in the cotton swab was around 11% of the total amount after the porcine skin surface was swabbed. The FITC-dextran was left on the porcine skin surface due to backflow of body fluid during attachment of the microneedles. As a result, the amount delivered into the skin was roughly 71% of the total amount coated on the microneedles. When 18% of the total formulation was left on the surface of microneedles, the amount of left protein on the surface is 1.4% of left formulation, and 30% of left protein is HA.

### Diffusion profile of fluorescence in skin

**Figure 4:**
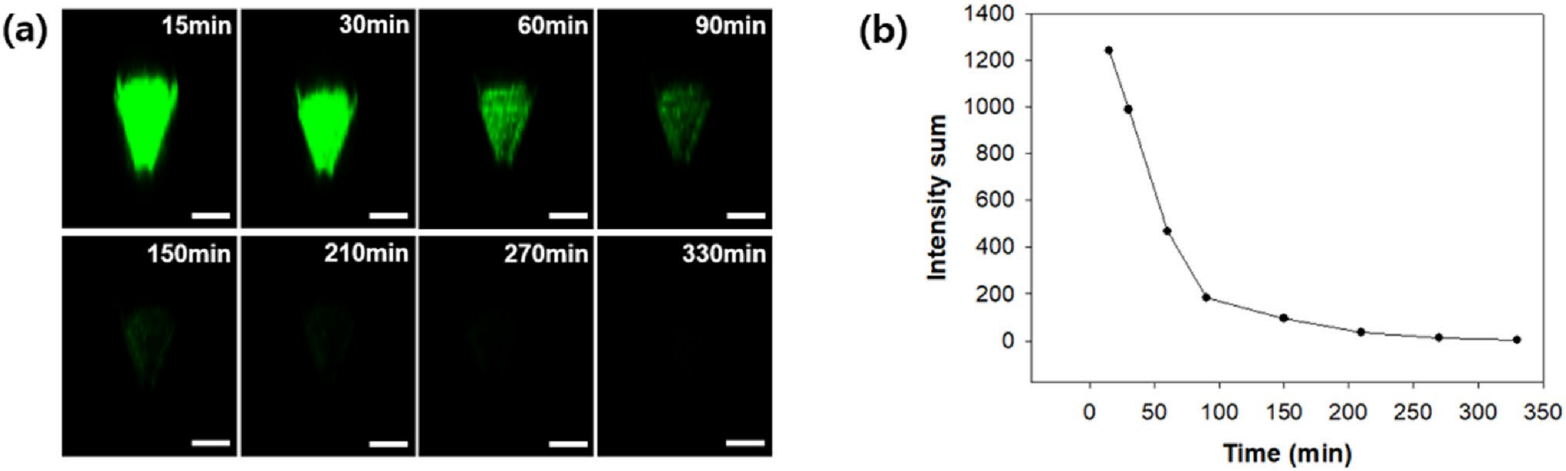
(**a**) Fluorescence image on the microneedle surface observed with a confocal microscope at 15, 30, 60, 90, 150, 210, 270, and 330 min (scale bar = 200 μm). (**b**) Change in fluorescence intensity on the surface of microneedle tips from 15 to 330 min.

A distribution of fluorescence intensity higher than the user-defined threshold intensity was observed from 15 to 330 min after initial administration (Fig. 4a). FITC-dextran was used as a model drug with a molecular weight (MW) of 70 kDa because the molecular weight of the antigen of H3N2 split vaccine is close to 70 kDa. In addition, changes in intensity on the surface of the microneedles were quantitatively expressed over time (Fig. 4b). The fluorescence intensity decreased most sharply between 30 and 60 min after initial administration. Most of the fluorescence signal was undetectable after 210 min. The diffusion rate of the FITC-dextran was affected by the phase of dissolution of the coating layer from a solid layer into a gel and then a liquid solution. The FITC-dextran slowly diffused in the skin over several hours as the dissolution of the coating layer progressed.

### Evaluation of hemagglutinin content and thermal stability measurement using HPLC and ELISA

The measured HA content of the liquid vaccine was 28% and 22% at 25 °C and 12% and 8% at 40 °C by HPLC and ELISA, respectively. Values of 31%, 41%, and 43% of HA content were obtained by ELISA after additional exposure of H3N2 Film, Film-Tre20, and Film-Tre100 at 40 °C for 24 h, respectively. HA contents of 23%, 49%, and 48% were detected by HPLC measurement for H3N2 Film, Film-Tre20, and Film-Tre100 after exposure at 40 °C for 24 h. Comparisons are summarized in Table 1. HPLC results were similar to those of ELISA results because ELISA and HPLC used the same pretreatment to obtain active HA. The ELISA assay and SRID are conventional methods for determining the HA content of vaccines. However, it takes 2–3 months to prepare standard antibodies, and antibody preparation is a key factor that delays vaccine development during a pandemic. In addition, antibody preparation causes delay in the development of microneedle vaccines that require precise HA measurement to determine proper formulation and stability. The preparation of H3N2 microneedle vaccines using HPLC-based assays enables rapid preparation and accurate loading of the H3N2 vaccine. Liquid chromatography-tandem mass spectrometry was used to quantify the HA content, and the mass spectrometer offered a high level of selectivity^31^. However, the mass spectrometer is not easy to use for controlling and inspecting the operation^32^. Using the UV detector and the mass spectrometer at the same time will be more efficient than using them independently. In this study, the solid formulation showed improved stability. Trehalose contributed to improved thermal stability, but changes in trehalose content produced no noticeable differences in stability.

**Table 1:** HA content measured by HPLC and ELISA by exposure at 25 °C and 40 °C for 24 h after 24 h of drying process at 25 °C.

	Temperature of exposure for 24 h (°C)	HA content (%) by ELISA	HA content (%) by HPLC
**H3N2 solid vaccine**			
Film	25	68	67
	40	31	23
Film-Tre20	25	92	91
	40	41	49
Film-Tre100	25	97	96
	40	43	48
H3N2 liquid vaccine	25	22	28
	40	8.2	12

### Immune response of H3N2 microneedle vaccines in mice

**Figure 5:**
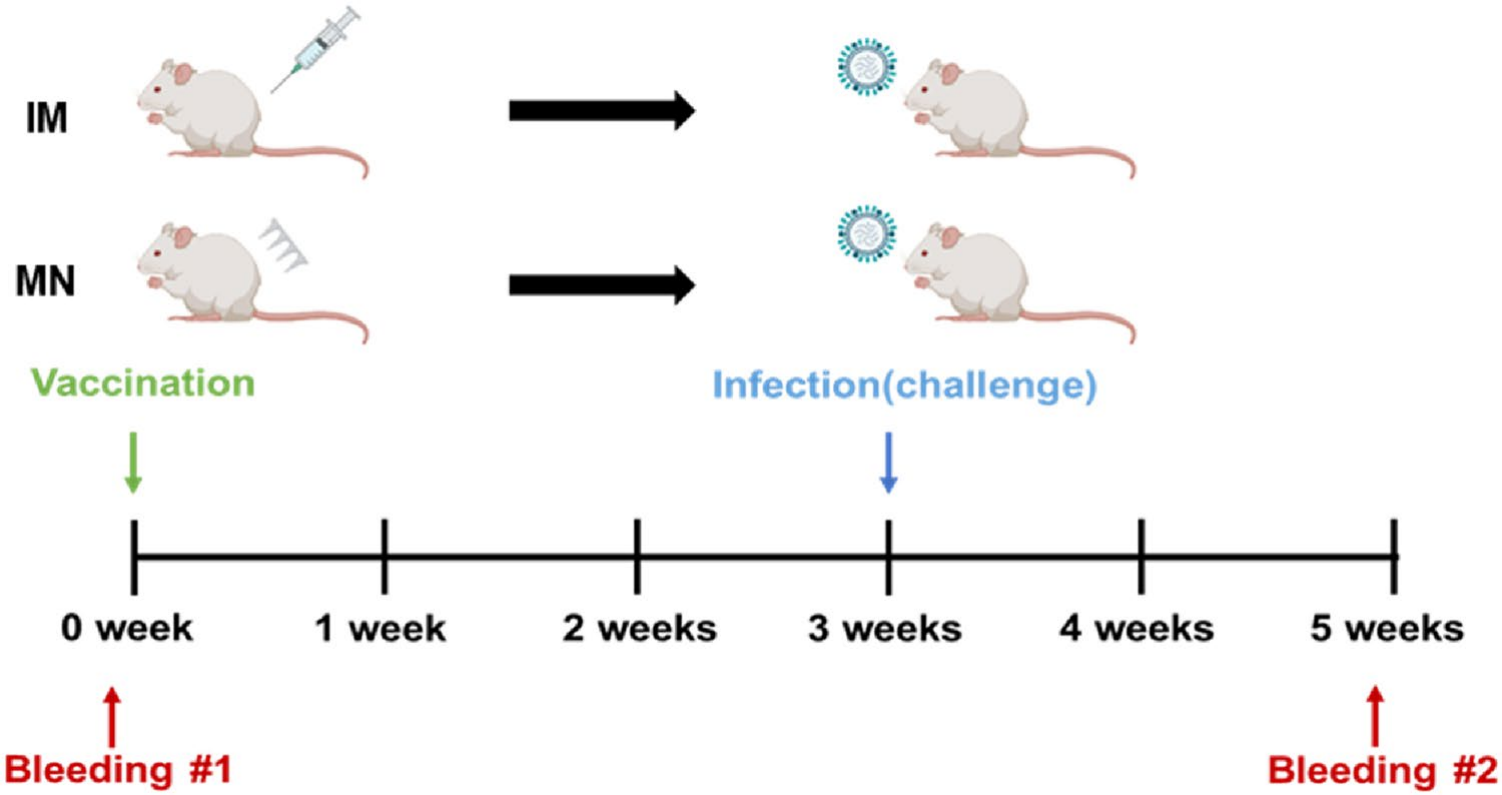
Brief schedule of i*n vivo* experiment. BALB/c mice were immunized intradermally with 19–20 A/KS/17 H3N2 microneedle vaccines (3 μg/mouse) or intramuscularly with vaccine formulations (3 μg/mouse). Mice were infected with the mouse-adapted 15–16 A/SW/13 H3N2 virus at 3 weeks after vaccination (50MLD_50_, 30 μl).

**Figure 6:**
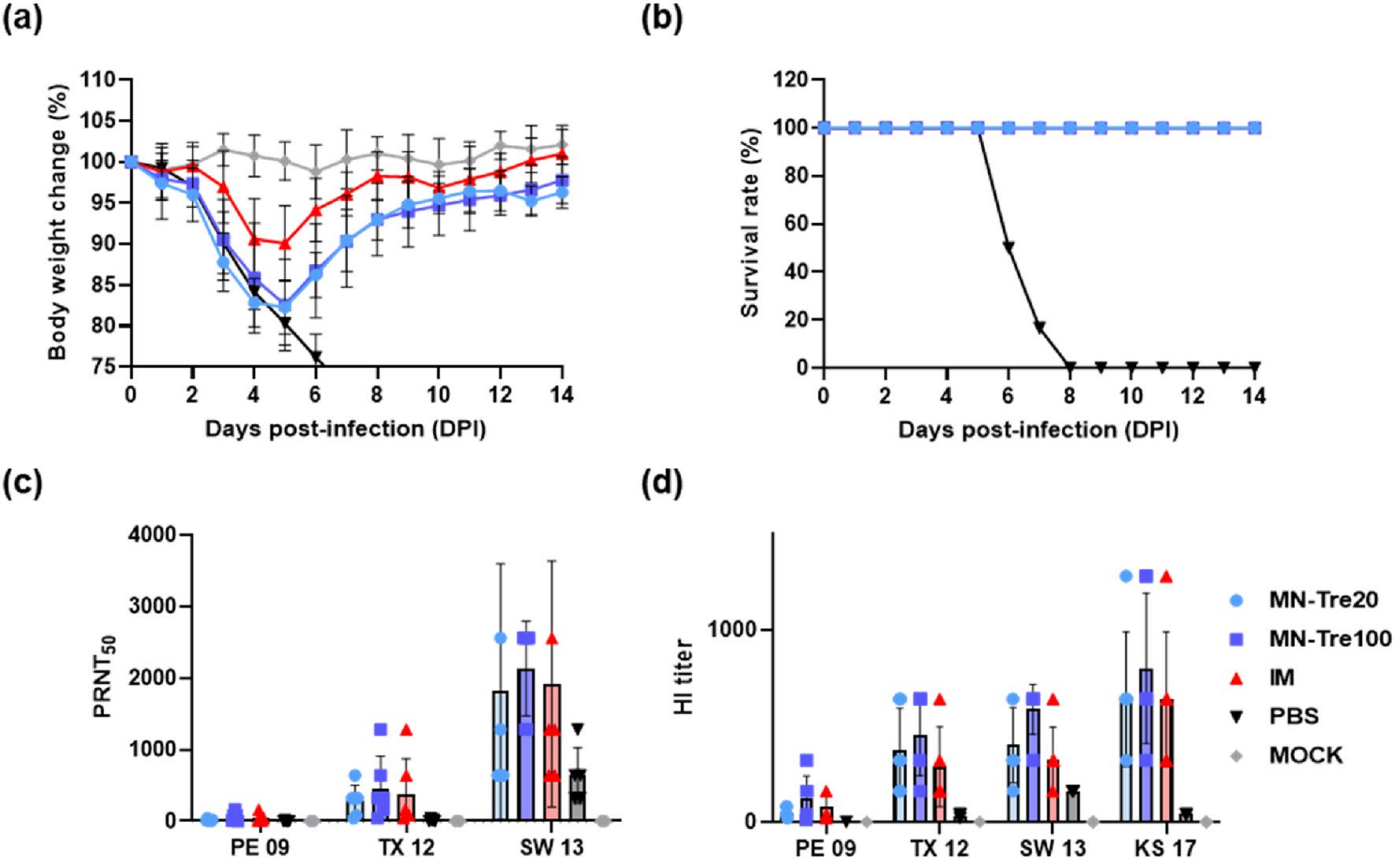
(**a**) Body weight change and (**b**) survival rate monitored for 14 days after challenge with mouse-adapted 15–16 A/SW/13 H3N2 virus in mice. MN-Tre20: Group administered transdermally with 20 times the amount of trehalose compared to the HA content of the vaccine, MN-Tre100: Group administered transdermally with 100 times the amount of trehalose compared to the HA content of the vaccine. IM: Group administered vaccine through intramuscular injection. (**c**) Neutralizing antibody titers of 19–20 A/KS/17 H3N2 vaccine microneedle and IM groups mouse sera at 14 days after challenge against A/PE/09 H3N2 virus, A/TX/12 H3N2, and A/SW/13 H3N2 virus. (**d**) Hemagglutination inhibition (HI) titers of 19–20 A/KS/17 H3N2 vaccine microneedle and IM groups mouse sera at 14 days after challenge against A/PE/09 H3N2 virus, A/TX/12 H3N2, and A/SW/13 H3N2 virus.

As shown in Fig. 5, BALB/c mice were immunized intramuscularly or transdermally with 19–20 A/KS/17 H3N2 vaccine. When the microneedle and IM groups immunized by 2019–20 seasonal H3N2 influenza (19–20 A/KS/17) vaccine were challenged with the mouse-adapted 2015–16 seasonal H3N2 influenza virus (15–16 A/SW/13), all microneedle and IM groups excluding the PBS group displayed complete cross-protection. Minor weight loss was observed in the microneedle group and the IM group in comparison to the MOCK group, but differences between the groups were not significant. In the PBS group, body weight declined by up to 70% of the initial weight (Fig. 6a). There was no statistically significant difference between the IM, MN-Tre20, and MN-Tre100 groups with respect to cross-protective response of the vaccine. In addition, although the epidemic periods of the 19–20 A/KS/17 and the 15–16 A/SW/13 viruses differ, they all belong to the same 3C.3a clade; thus, variations in antigenicity were not significant, resulting in a sufficient cross-protective immune response. In another study, when the influenza vaccine was delivered intradermally using microneedles, microneedle patches induced superior influenza-specific functional antibody titers, and improves the Th1 responses^33^.

### Cross-protective immune responses of H3N2 microneedle vaccines

In the case of PRNT analysis of the 14 days post-infection (DPI) mouse serum, neutralizing antibody titers of the IM and microneedle groups vaccinated with the 19–20 A/KS/17 H3N2 microneedle vaccines were higher than in the PBS group (Fig. 6c), which corresponded with our results of body weight loss and survival rate (Fig. 6a,b). Also, the neutralizing antibody titers of the microneedle group and the IM group in general were not statistically different. As a result of the hemagglutination inhibition (HI) assay, it was confirmed that the antibody titer was lower than the PRNT results. However, it showed a tendency similar to the PRNT result that the HI titer of MN-Tre100 was the highest compared with other groups (Fig. 6c,d).

The 2010–12 seasonal H3N2 influenza virus (10–12 A/PE/09), the 2014–15 seasonal H3N2 influenza virus (14–15 A/TX/12), and the 15–16 A/SW/13 belong to 1 clade, 3C.1 clade, and 3C.3a clade, respectively. The 19–20 A/KS/17 H3N2 microneedle vaccine (3C.3a clade) showed low immunogenicity against the 10–12 A/PE/09 and the 14–15 A/TX/12 due to their antigenic distance. Although the epidemic periods of the 19–20 A/KS/17 and the 15–16 A/SW/13 were different, they belong to the same 3C.3a clade. Thus, the cross-protective immune responses of these 3C.3a microneedle and IM groups were higher when compared to that of other viruses. Both microneedle and IM administration of the H3N2 vaccine showed comparable cross-protective immune responses. Split influenza vaccine in this study also contains NP apart from HA. Influenza NP induces antibodies as well as CTL which play a role in cross-protection^34^. However, a recent study revealed that more than 80% of plasmablasts induced in response to the influenza split vaccine are HA-specific, 1–2% of those are NA specific and the remaining cells have other targets, such as NP and M^35^. It suggests that the immune response to HA dominates the majority of immune response to the influenza split vaccine.

## Conclusions

HA content measured by HPLC analysis was comparable to that measured using ELISA, and H3N2 microneedle vaccines could be prepared without the use of a standard antibody. The use of solid vaccines displayed greater thermal stability than the conventional liquid vaccines when comparing the solid H3N2 microneedle vaccine formulation to the liquid H3N2 vaccine. The H3N2 microneedle vaccine formulation was successfully administered intradermally, and 71% of the coating layer was dissolved within 30 min, with the vaccine rapidly diffusing into the skin. Administration of the 19–20 A/KS/17 microneedle vaccine elicited cross-protection comparable to the results of IM administration according to survival rate and body weight changes in mice. Both the microneedle and IM groups showed cross-protective immune responses against the mouse-adapted 15–16 A/SW/13 virus. With regards to cross-protection efficacy, antigenic distance proved to be a more important variable than route of administration. The 19–20 A/KS/17 microneedle vaccine provided excellent immunity against the 15–16 A/SW/13 as it is in the same clade as the 19–20 A/KS/17, whereas the 10–12 A/PE/09 and the 14–15 A/TX/12 had relatively low cross-protective immune responses most likely due to their antigenic distance. Overall, the use of HPLC may prove to be an effective method for rapid preparation of seasonal H3N2 influenza microneedle vaccines as well as being an alternative solution to combat antigenic variants.

## Methods

### Conventional influenza vaccine

The 19–20 A/KS/17 mono-valent H3N2 seasonal vaccine was obtained from Il-Yang Pharmaceutical Co., Ltd. (Yongin, Republic of Korea). The vaccine solution consists of phosphate-buffered saline (PBS) and H3N2 vaccine antigen, and the HA content of the vaccine was 30%.; there were no other additives in solution. PBS and trehalose were purchased from Sigma-Aldrich (St. Louis, MO, USA). Polylactic acid (PLA) was purchased from Lactel (Birmingham, AL). Carboxymethyl cellulose (CMC) was purchased from Howon (Gyeonggi-do, South Korea). Porcine skin was purchased from Cronex (Seongnam, Korea).

### Preparation of H3N2 microneedle vaccine

The master mold of the microneedles was manufactured by the micromilling method. The master mold was filled with a 10:1 mixture of poly-di-methylsiloxane (PDMS) and a curing agent, and after air bubbles were removed in a vacuum chamber, the PDMS was cured in an oven at 70 °C for 1 h. PLA microneedles were manufactured by placing PLA pellets on the cured PDMS mold, melting them in an oven at 190 °C (VOS-301, EYELA, Tokyo). The coating solution was composed of CMC and trehalose. The 19–20 A/KS/17 H3N2 vaccine solution was concentrated to 2 mg/ml using an Amicon Ultra-0.5 Centrifugal filter device (Amicon Ultra 10 K, Billerica, MA) and a microcentrifuge (GYROZEN, Gimpo, Korea). The amount of trehalose was 20 or 100 times that of HA to assess the efficacy of stabilizer, and the concentration of CMC in the final coating solution was 6% (w/w). The coating solution was loaded in a 600 μm deep coating well, and the H3N2 formulation was applied to the uncoated PLA MNS using the dip-coating method. The composition of two solid formulations with trehalose was: H3N2:trehalose:CMC = 1:20:50 and 1:100:50, respectively. The amount of H3N2 loaded per microneedle array patch was measured by the Bradford protein assay after putting the coated microneedles in 1 ml of PBS and dissolving the H3N2 formulation for 30 min (*n* = 5). The detailed morphology of the coated microneedles and the uniformity of the coating amount over the array were also observed using a scanning electron microscope (SEM, JSM-7001F, JEOL, Ltd, Tokyo, Japan).

### Puncture performance of microneedles for observation of successful insertion

Trypan blue microneedles were prepared using the same coating method as that for the H3N2 microneedle vaccines. The trypan blue-coated microneedle array patch was applied to the full porcine skin (2 × 2 cm, about 5 mm thick) with force of 4.5–5 kg for 60 s. The number of blue dots was counted after imaging the porcine skin surface using a stereo microscope (Leica THUNDER model organism imaging system, Wetzlar, Germany). Puncture performance is the ratio of the number of blue holes to the total number of microneedles. The porcine skin was frozen with liquid nitrogen, and the blue dots were cut with a microtome. The image of cross-sections was taken with a stereo microscope.

### Delivery efficiency of H3N2 microneedles for measurement of predetermined dose of vaccine

In order to measure the amount of the coated layer with H3N2 vaccine delivered into the skin, microneedles were coated with a formulation containing the model drug FITC-dextran (MW: 70 kDa) (Sigma-Aldrich, St. Louis, MO, USA) and they were attached to porcine skin at 37 °C for 30 min. After the microneedles were removed, the insertion site was gently wiped with a dry cotton swab. To quantify the amount of FITC-dextran on the skin surface, the microneedles and the swab were placed in 1 ml of PBS separately overnight. The amount of FITC-dextran was measured by comparison with a calibration curve at 490 nm of excitation wavelength and 520 nm of emission wavelength using a fluorescence spectrophotometer (Multi-label Plate Reader, PerkinElmer, Boston, USA).

### Dissolution of coating layer and diffusion of model drug in skin

To quantify the diffusion profile of H3N2 in the skin over time, FITC-dextran (MW: 70 kDa) was used as a model drug with a molecular weight similar to that of the H3N2 antigen (MW: 75 kDa). At 15, 30, 60, 90, 120, 150, 180, 210, 240, 270, 300, and 330 min after microneedle administration, the distribution of fluorescence of FITC-dextran in the skin was observed using a confocal microscope (Ex: 490 nm/Em:520 nm, ECLIPSE TE2000-E, Nikon, Tokyo, Japan). Sequential z-stack images were captured at 15-μm-depth intervals from the skin surface to a depth of 840 μm. Analysis was performed using a plot profile of Image J (National Institutes of Health, Bethesda, MD) of fluorescent signal intensity in confocal z-stacks obtained along the inserted microneedles.

### Measurement of HA content of H3N2 vaccines using HPLC

The liquid vaccine was placed at 4 °C or at 40 °C for 24 h. The coating solution with the same composition as the coating layer was dried at 25 °C for 24 h to obtain the dried film and then the dried film was placed at 4 °C or 40 °C for 24 h. After exposure at two temperature, the solidified film was redissolved and the HA content was measured by ELISA and HPLC (Table 2). The thermal stability of the solidified 19–20 A/KS/17 H3N2 microneedle vaccine was evaluated. Since a large amount of sample was required for accurate analysis of HA content by repeated experiments, H3N2 vaccine film was prepared by spreading coating vaccine solution on the PLA disk^11^. Three types of H3N2 vaccine film were prepared from three kinds of solutions. The composition of the three H3N2 coating solutions is summarized in Table 2. The three solutions (each 166 μl) were dropped on three separates flat 1 cm^2^ PLA disks and spread over each disk. Solidified film was obtained by drying the coating solutions at 25 °C for 24 h. Then the thermal stability of the H3N2 microneedle vaccines was determined by storing the samples at 4 °C or 40 °C for 24 h (Table 1). Samples were dissolved in PBS at 4 °C overnight for measurement of HA content. 4 °C stored H3N2 liquid vaccine was positive control. To set the calibration curve, the 19–20 A/KS/17 H3N2 vaccine was diluted with PBS to prepare standards with the range of 56 to 449 μg/ml of concentration. A zwittergent 3–14 (Sigma-Aldrich, St. Louis, MO, USA) solution (10% w/w) was added to standard and sample solutions to achieve a 1% concentration of zwittergent in the mixture. Then TPCK-trypsin (Thermo-Fisher, Waltham, MA) was added and the mixture was incubated at 37 °C for 3 h. After centrifugation (1000*g*, 4 °C, 5 min) using a microcentrifuge (GYROZEN, Gimpo, Korea), the supernatant was harvested and the remnant was removed. Then 500 mM of dithiothreitol (DTT) was added to the solution for a 25 mM DTT in the mixture, and then the mixture was maintained at 90 °C for 5 min. The active HA1 protein peak was selectively obtained by analyzing the pretreated samples by reverse phase-liquid chromatography (RP-HPLC, Agilent 1200 series, Agilent Technologies, Santa Clara, CA, USA). Column Poros R1/10 (2.1 mm × 100 mm) was maintained at 65 °C and samples were stored at 4 °C before injection. Mobile phase A was 0.1% trifluoroacetic acid and 5% acetonitrile in water, and mobile phase B was 0.1% trifluoroacetic acid and 25% methanol in acetonitrile. The flow rate was 0.8 ml/min, and the total run time was set to 25 min. The injection volume of the sample was 40 μl and the sample was detected at 214 nm by UV detector^36–38^.

**Table 2:** Information on H3N2 vaccine films for stability test using RP-HPLC and ELISA analysis.

	Composition (vaccine, CMC, Trehalose, PBS total 100%)		Conditions for stability test	
	Before drying	After drying	Drying	Additional exposure
**H3N2 solid vaccine**				
Film	0.12, 6, 0, 93.88	1.96, 98.04, 0, 0	25 °C at 24 h	4 °C for 24 h 40 °C for 24 h
Film-Tre20	0.12, 2.4, 6, 91.48	1.41, 70.39, 28.2, 0		
Film-Tre100	0.12, 12, 6, 81.88	0.66, 33.14, 66.2, 0		
H3N2 liquid vaccine	N/A	N/A	N/A	4 °C for 24 h 40 °C for 24 h

### Measurement of HA content of H3N2 vaccines using ELISA

A 10% (w/v) solution of zwittergent 3–14 was added to the sample, and the mixture was stored at 25 °C for 30 min. After the pretreatment process was completed, 100 μl of the 19–20 A/KS/17 H3N2 vaccine was applied to a Nunc-Immuno™ MicroWell™ 96 well solid plate (Sigma-Aldrich, St. Louis, MO) and samples were stored at 4 °C overnight. Group 2 IAV^27^ universal antibody 4F11 was used as the primary antibody, and HRP-conjugated goat anti mouse IgG (Southern Biotechnology, Birmingham, AL) was used as the secondary antibody. The absorbance of the plate was measured at 450 nm using a Synergy H1 hybrid multimode microplate reader (BioTek Instruments Inc, Winooski, VT)^39^.

### In vivo animal experiment

All experiments were approved by the Institutional Animal Care and Use Committee of Korea University (KOREA-2018-0057) and conducted in accordance with the Korea University Guidelines, which are based on the National Institutes of Health’s Guide for the Care and Use of Laboratory Animals, and with the ARRIVE guidelines. Female BALB/c mice (4–6 weeks old; Orient Bio, Seongnam, Republic of Korea) were vaccinated using the 19–20 A/KS/17 H3N2 microneedle vaccines based on the schedule (Fig. 5). After hair was removed from the backs of the mice, microneedle vaccines were administered for 10 s and fixed using a clamp for an additional 30 min. At 3 weeks after vaccination, the mouse-adapted 15–16 A/SW/13 H3N2 virus was challenged through the nasal cavity (50MLD_50_, 30 μl). Weight change and survival rate were checked daily for 14 days after the challenge inoculation^40,41^. Mice who lost 25% or more of their original body weight were considered experimentally dead and were euthanized. PBS was administered intramuscularly to a negative control group. To evaluate cross-reactivity by the HI assay and the PRNT test, mouse serum was collected at 14 days after mouse challenge inoculation with the virus and the test was performed^42,43^. The 19–20 A/KS/17 H3N2 vaccine (3C.3a clade) was used for vaccination. A three season virus was used for the PRNT test. The 10–12 A/PE/09 H3N2 virus virus (NIBSC code: 09/208) used for PRNT is 1 clade, the 14–15 A/TX/12 H3N2 virus (NIBSC code: 12/298) is 3C.1 clade, and the 15–16 A/SW/13 H3N2 virus (obtained from KCDC) virus is 3C.3a clade. After reacting with the same amount of virus as the serum obtained from the mouse groups, the cells were infected and the maximum dilution factor that reduced plaque formation by 50% or more was compared to the maximum dilution factor of the control group. Serum was also obtained from unimmunized mice that were established as a MOCK group (Table 3).

**Table 3:** Information on challenge test using 19–20 A/KS/17 H3N2 microneedle vaccines against different-year H3N2 viruses using the measurements of body weight change, survival rate, and PRNT test. *MNs* microneedles, *IM* intramuscular administration, *PRNT* plaque reduction neutralization test.

	MNs		IM	PBS	MOCK
	MN-Tre20	MN-Tre100			
**Dose (μg)**					
19–20 A/KS/17 H3N2 vaccine	3	3	3	–	–
**Challenge**					
15–16 A/SW/13 H3N2 virus	Y	Y	Y	Y	–
**PRNT**					
10–12 A/PE/09 H3N2 virus 14–15 A/TX/12 H3N2 virus 15–16 A/SW/13 H3N2 virus	Y	Y	Y	Y	–

### Statistical method

A two-tailed Student's *t* test (*α* = 0.05) was performed when comparing two different conditions, and ANOVA was used when comparing multiple groups. Values of *p* < 0.05 were considered statistically significant.

## Data Availability

The datasets generated during and/or analyzed during the current study are available from the corresponding author on reasonable request.
