# Author Correction: Development of the H3N2 influenza microneedle vaccine for cross-protection against antigenic variants

**DOI:** 10.1038/s41598-022-20913-1

**Published:** 2022-10-03

**Authors:** Yura Shin, Jeonghun Kim, Jong Hyeon Seok, Heedo Park, Hye-Ran Cha, Si Hwan Ko, Jae Myun Lee, Man-Seong Park, Jung-Hwan Park

**Affiliations:** 1 Department of BioNano Technology, Gachon University, Seongnam, Republic of Korea; 2 Department of Microbiology, Institute for Viral Diseases, Chung Mong-Koo Vaccine Innovation Center, College of Medicine, Korea University, 73 Goryeodae-ro, Seongbuk-gu, Seoul, 02841 Republic of Korea; 3 Department of Microbiology and Immunology, Institute for Immunology and Immunological Diseases, Yonsei University College of Medicine, Seoul, 03722 Korea

Correction to: *Scientific Reports* 10.1038/s41598-022-16365-2, published online 16 July 2022

In the original version of this Article, Jung-Hwan Park was incorrectly affiliated with ‘QuadMedicine R&D Centre, QuadMedicine Co., Ltd, Seongnam, Republic of Korea’. The correct affiliation is listed below.

Department of BioNano Technology, Gachon University, Seongnam, Republic of Korea

The original Article has been corrected.

